# Genetic variants of *SLC12A3* modulate serum lipid profiles in a group of Mongolian pedigree population

**DOI:** 10.1186/s12944-018-0737-1

**Published:** 2018-04-16

**Authors:** Caiyan An, Junqing Liang, Kejin Zhang, Xiulan Su

**Affiliations:** 1 Clinical Research Center of the Affiliated Hospital, Inner Mongolia Medical University, Hohhot, 010050 Inner Mongolia China; 2 Key Laboratory of Resource Biology and Biotechnology in Western China (Ministry of Education), College of Life Science, Institute of Population and Health, Northwest University, Xi’an, China; 3 Department of Galactophore, Affiliated People’s Hospital of Inner Mongolia Medical University, Hohhot, Inner Mongolia China

**Keywords:** Genetic variants, Low-density lipoprotein cholesterol (LDL-C), Family- based association test (FBAT), Mongolian

## Abstract

**Background:**

The serum lipid profile, including LDL-C level, is associated with hypertension which is the major cause of cerebrovascular disease (CVD) amounting 30% of global death rate. Previous work also demonstrated important roles of genetic variants of *SLC12A3* gene on human CVD, hypertension and other diseases in Mongolian population. However, the relationship between *SLC12A3* gene polymorphisms on individuals’ lipid profile is still unknown.

**Methods:**

A panel of 15 SNPs of *SLC12A3* gene was genotyped within a 424 Mongolians pedigree cohort. The associations between *SLC12A3* polymorphisms and four lipid profiles were analyzed by family-based association test (FBAT) and confirmed with haplotype analysis.

**Results:**

From both single site and haplotype analyses, the results demonstrated a close relationship between *SLC12A3* polymorphisms and LDL-C level. Two SNPs, rs5803 and rs711746 showed significant associations with individuals’ serum LDL-C level (*z* = − 2.08, *P*_*-e*_ = 0.038; *z* = 2.09, *P*_*-e*_ = 0.023, respectively), and distribution of haplotypes constructed by two SNPs also associated with participants’ serum LDL-C level, significantly (Global *Chi*^2^ = 9.06 *df* = 3, *P* = 0.028).

**Conclusion:**

Our results demonstrated the importance of *SLC12A3* polymorphisms in individuals’ difference about their serum lipid profiles, thereby providing evidence that the genetic variants may contribute to CVD development via modulating person’s LDL-C level and blood pressure, in certain contexts.

**Electronic supplementary material:**

The online version of this article (10.1186/s12944-018-0737-1) contains supplementary material, which is available to authorized users.

## Background

Lipid metabolism plays a role in the development of common chronic human diseases, including cerebrovascular disease(CVD)and other diseases [1]. Parameters to profile the lipid metabolism, which includes the level of total plasma cholesterol (TCHO), triglycerides (TG), low-density lipoprotein cholesterol (LDL-C), and high-density lipoprotein cholesterol (HDL-C), are of great importance, and often determined to estimate the risk of CVD [2]. In large epidemiological studies, alteration of these parameters in serum, such as increased levels of TG, LDL-C, and decreased levels of HDL-C, have been identified to be crucial indicative factors to the pathogenesis of CVD [3, 4], and even were suggested as targets for therapeutic intervention [5].

Serum lipid levels are highly heritable and studies consistently estimated that over 50% of the total inter-individual variation in serum lipid levels can be explained by genetic variants [6], that is, the bulk of lipoprotein genetics may be contribute to the various of lipoprotein traits [7], to our knowledge. Increasing evidences from GWAS studies and association tests suggested *SLC12A3* gene which encodes Na-Cl cotransporter, as a candidate gene for human cardiovascular disease via affecting blood pressure [8]. Asselbergs et al., [9] confirms the association between *SLC12A3* variants and individuals’ HDL-C levels, which may lead to CVD, in a large-scale meta-analysis related to 66,240 individuals of European ancestry. Our previous work also demonstrated a close association between *SLC12A3* polymorphism and human hypertension in Mongolians, both within random and pedigree populations [10, 11]. Therefore, we hypothesized that *SLC12A3* gene might influence individuals’ serum lipid levels, modify their blood pressure, and increase the risk of CVD or related diseases in Mongolians and the aim of this study is to certify the relationship between genetic variants of *SLC12A3* and individual’s serum lipid levels in Mongolians.

In the present study, we have conducted a family-based study to test the association between a panel of 15 SNPs within *SLC12A3* gene and four measurable serum lipid levels, and confirmed that the genetic variants of *SLC12A3* may contribute to the variation of serum lipid profiles among individuals, in a group of Mongolian pedigree population.

## Methods

### Subjects

The probands of family-based samples in this study were recruited from the Affiliated Hospital of Inner Mongolia Medical University, through electronic medical chart review. All participants were of Mongolian ethnic origin and from Inner Mongolia Autonomous Region of China. When TCHO is within 2.8~ 6.5 mmol/L, TG within 0.56~ 1.70 mmol/L, HDL-C within 1.03~ 2.07mmoL/L and LDL-C within 1.0~ 4.4 mmol/L, we diagnosed the subject as normal. Subjects with one or more of the indicators above the normal range were identified as dyslipidemias. The relatives of the probands were tracked and diagnosed based on the same criteria. Probands with history of CVD such as coronary artery disease (CAD) and stroke, diabetes, hyper- or hypo-thyroids, hypertension, and chronic renal diseaseas well as their relatives were excluded. All participants were free from medications known to affect serum lipid levels before blood collection. Totally, there were 424 participants involved within 100 hundred extended families, including 139 (F90) parental group, 174 (F76) of offspring group and 111 (F47) relatives. All of them had available variables about their personal information and serum lipid profiles. Written informed consents were obtained from all participants. The study was performed in accordance with the Declaration of Helsinki, and approved by the Ethical Committee of Affiliated Hospital of Inner Mongolia Medical University.

### Demographic information and serum lipid parameters

In this study, demographic variables collected included age, gender. Subjects’ height and weight were measured, BMI (body mass index) was calculated as weight (kg) divided by the square of height (m), and WHR (waist-hip ratio) was also assessed by dividing waistline (cm) by hipline (cm). Normal weight, overweight, moderate obesity and severe obesity were defined by BMI and WHR indexes according with WHO recommendation. Blood pressure was measured three times, with a 2-min interval between each measurement. SBP was recorded to the nearest 2 mmHg at the appearance of the first Korotkoff sound (phase I), and DBP was recorded to the nearest 2 mmHg at the disappearance of the fifth Korotkoff sound (phase V). The SBP and DB*P* values were calculated as the means of three consecutive physician-obtained measurements. In this study, we also checked two health behaviors, including smoking and drinking. Smoking was defined as smoking at least one cigarette per day for at least one year, and drinking was defined as consuming 50 g or more alcohol per day for at least one year.

Blood samples were obtained from the antecubital vein after ≥8 h of fasting. A part of samples were collected and used to determine serum lipid levels. Another part of samples were transferred into a tube with anti-coagulate solution and used to extract deoxyribonucleic acid (DNA). To profile individuals’ serum lipid status, four important parameters, including total plasma cholesterol (TCHO), triglycerides (TG), high-density lipoprotein cholesterol (HDL-C), and low-density lipoprotein cholesterol (LDL-C), were measured within 8 h, using routine methods. Genomic DNA was isolated from peripheral blood leukocytes using an AxyPrep-96 DNA Extraction Kit (Axygen, Union City, CA, USA).

### Tagger SNP selection and genotyping

The tagger SNPs of *SLC12A3* gene were designed, chosen and genotyped among this pedigree sample, as described previously [10]. Briefly, 15 SNPs of the *SLC12A3* gene were selected in this study, including rs4784733, rs2304478, rs13306673, rs2289119, rs8043560, rs2304483, rs5803, rs7187932, rs6499858, rs11644728, rs8049280, rs7204044, rs2010501, rs2399594 and rs711746. Genomic DNA was obtained from 1 mL venous blood using the AxyPrep-96 DNA Extraction Kit (Axygen, Union City, CA, USA), and subjected to multiplex PCR-ligase detection. Then, the PCR product was mixed with equal volume fluorescent molecular weight standards (Applied Biosystems, Foster City, CA, USA) and deionized formamide, denatured for 2 minat 95 °C, and then electrophoresized in 5% polyacrylamide gels containing 5 mol/L ureophil. Each subject’s SNPs genotypes were estimated with GENESCANTM 672 software, and analyzed with GeneMapper 3.0. Strict quality control measures were implemented with more than 99% concordance in duplicate randomly selected samples.

### Statistical analysis

Four serum lipid variables (TCHO, TG, HDL-C, and LDL-C),which can be used to assess individuals’ metabolism status, were selected as quantitative phenotypes for association analysis. PASW statistics 18 (formerly SPSS Statistics; http://www.spss.com.hk/statistics) was used to analyze the characteristics of demographic variables and the features of sample’s serum lipid levels with parental group. The difference of continuous variables (e.g., age, BMI, WHR, TCHO, TG, HDL-C, LDL-C) were analyzed by t test for two groups and ANOVA for three or more groups, and non-parameter test (e.g., Kruskal Wallis test) also performed if variables didn’t meet the normal distribution. The categorical variables (e.g., smoking and drinking) were analyzed by Fisher’s exact Chi-square test. The correlation of demographic variables was evaluated by multiple regression analysis, within parental group. To identify potential Mendelian errors, the PEDCHECK program was used to test the family structure before running the family-based association test [12]. The pedigrees, which were not consistent with Mendelian inheritance, were excluded from quantitative phenotypic analysis. For single marker, it was analyzed by FBAT program in the additive and dominant models, respectively [13], with a null hypothesis of no linkage and no association. Then, the haplotypes created based upon significant association with the individuals’ serum lipid level in both additive and dominant genetic models, were tested using the HBAT extension of FBAT. Both haplotype-specific and a multiallelic (global) test were calculated for haplotypes for which there were a minimum of 10 informative families. The empirical variance estimator option (*−e*) was used as our primary hypothesis, and *P*-values generated by permutation tests (default *n* = 10,000) as correction for multiple testing. The statistical power of this study with the present sample size was also estimated by Quanto 1.2.4 [14], under the “case-parent trio” type of study designs, and with a rather lower genetic effect (*R*_g_^2^ = 0.10) for all SNPs. All testing was performed with a type I error rate of 0.05.

## Results

### Demographics and phenotype values in participants

Table 1 described the feature of sample and its subgroups, and the difference among them. Parental group had a higher average age than that of total or offspring group (*P* <  0.001), as our supposed. And no any difference about BMI, WHR and four lipid levels’ variable, was found out. Furthermore, in parental group, the gender difference analysis indicated that, male had a higher WHR value and lower HDL-C level than that of female (0.91 ± 0.05 vs. 0.87 ± 0.06, *t* = 2.44, *P* = 0.019; and 1.26 ± 0.38 mmol/L vs. 1.43 ± 0.34 mmol/L, *t* = − 2.81, *P* = 0.006, respectively). Consistent with Chinese traditional concept on marriage [15], male was older than female in parental group (56.82 ± 12.76 vs. 50.97 ± 16.02, *t* = 2.20, *P* = 0.029).

**Table 1:** The demographic and baseline lipid levels in serum

Variables	Total (*n* = 424)	Parental (*n* = 139)	Offspring (*n* = 174)	*t* test	
				Statistic values	*P* values
Age	46.40 ± 15.80	53.03 ± 15.16	41.10 ± 14.26	*t* = 7.15	< 0.001
BMI	25.18 ± 5.03	25.43 ± 5.18	24.98 ± 4.91	*t* = 0.49	0.626
WHR	0.88 ± 0.06	0.88 ± 0.06	0.88 ± 0.06	*t* = − 0.23	0.816
TCHO (mmol/L)	4.61 ± 1.53	4.58 ± 1.39	4.64 ± 1.64	*t* = − 0.31	0.761
TG (mmol/L)	1.52 ± 1.86	1.45 ± 1.73	1.58 ± 1.97	*t* = − 0.63	0.530
HDL-C (mmol/L)	1.35 ± 0.37	1.37 ± 0.36	1.34 ± 0.38	*t* = − 0.76	0.445
LDL-C (mmol/L)	3.03 ± 1.13	3.00 ± 1.06	3.07 ± 1.19	*t* = − 0.54	0.586

*Abbreviations*: *TCHO* total plasma cholesterol, *TG* triglycerides, *HDL-C* high-density lipoprotein cholesterol, *LDL-C* low-density lipoprotein cholesterol, *BMI* body mass index, *WHR* waist-hip ratio

The effects of non-genetic variants on individuals’ serum lipid levels were estimated by *t*-tests within parental group. The gender, age (median = 54 years), BMI (median = 24.93) and WHR (median = 0.89) were all looked as categorical variables and analyzed in parental group. Female had a higher HDL-C level than that of male (*t* = − 2.81, *P* = 0.006). Subjects’ age showed an important role in HDL-C and LDL-C levels, the older had a lower HDL-C level and higher LDL-C level (*t* = − 2.01, *P* = 0.046 for HDL-C; *t* = − 2.18, *P* = 0.030 for LDL-C, respectively). The fitness status of subject, BMI index, also showed a close relationship with their serum lipid profiles (*t* = 3.06, *P* = 0.003 for TCHO; *t* = 2.61, *P* = 0.011 for TG and *t* = 4.16, *P* <  0.001 for LDL-C, respectively).

### Single SNP association analysis

Table 2 demonstrated a consistent association between genetic polymorphisms of *SLC12A3* gene and individual s’ LDL-C level, which has been found in our previous case-control study [16], and further confirmed by FBAT analyses within the present family-based Mongolian pedigree sample. Firstly, four SNPs, rs2304478, rs5803, rs7204044 and rs711746 showed significant associations with individuals’ serum LDL-C levels (all *P*s < 0.05). Then rs5803 and rs711746 sites still demonstrated a close relationship with LDL-C, using the empirical variance estimator option (−*e*) EM algorithm, both under the additive and dominant FBAT models (z = − 2.08, *P* = 0.037, *P*-e = 0.038 for rs5803, and z = 2.09, *P* = 0.036, *P*-e = 0.023 for rs711746 with dominant model). Meanwhile, both two SNPs also demonstrated a significant association with individuals’ serum TCHO and TG level (Additional file 1: Table S1).

**Table 2:** The association between tagger SNPs of *SLC12A3* and LDL-C level by FBAT method

Marker^a^	Allele and frequency^b^		FBAT test statistics		*P*	*P*_*-e*_^d^
	Allele	Freq.	*Var* (*S*)	*z* ^c^		
*Additive model*						
rs2304478	G:A	0.14	134.08	−2.47	0.014	–
rs5803	T:C	0.26	143.90	−2.06	0.039	0.022
rs7204044	G:A	0.11	102.28	−1.99	0.047	–
rs711746	G:A	0.45	197.57	2.22	0.027	0.039
*Dominant model*						
rs2304478	G:A	0.14	93.68	−2.07	0.038	–
rs5803	T:C	0.26	84.56	−2.08	0.037	0.038
rs711746	G:A	0.45	103.24	2.09	0.036	0.023

*Abbreviations*: *FBAT* Family Based Association Test with the additive model, *Freq.* frequency of allele, *Var (S)* is a matrix, calculated under the null and used to standardize S

^a^Markers, which show significant association were demonstrated in this table; ^b^alleles detected in more than 10 informative families; and the lower frequency allele was used in following statistics calculation. ^c^z-score calculated based on a biallelic marker model, positive z values and *p* < 0.05 indicated a high-risk haplotype; ^d^*p*_e_, significance test by FBAT with empirical variance estimator option [*−e*], non-significant markers displayed with hyphen (−)

**Fig. 1:**
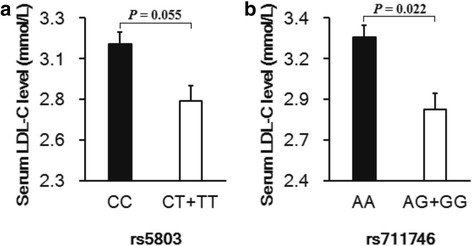
Impacts of rs5803 and rs711746 polymorphisms on individuals’ LDL-C level, under dominant model. **a** For rs5803, individuals with the CC genotype were more likely to have a higher LDL-C level that the individuals with the CT + TT genotype; (**b**) For SNP rs711746, individuals with the AA genotype were more likely to be a higher LDL-C level that individuals with the AG + GG genotype

Within parental group, comparison about the average of serum lipid levels among different genotypes, also confirmed the close relationship between two SNPs (rs5803 and rs711746) and individuals’ serum LDL-C levels. The genotype CC of rs5803 had a higher LDL-C level than that of allele T carriers (3.14 mmol/L vs. 2.79 mmol/L, *F* = 0.804, *sig.* = 0.372, *t* = − 1.94 *df* = 131, *P* = 0.055), while the difference did not reach statistical significance (Fig. 1a). While between AA genotype and allele G carrier of rs711746, it had a significant difference for their LDL-C levels (3.28 mmol/L vs. 2.84 mmol/L, *F* = 0.011, *sig.* = 0.918, *t* = − 2.31 *df* = 127, *P* = 0.022) (Fig. 1b). However, no similar results/trends were found out for serum TCHO and TG levels. It suggested that the CC genotype of rs5803 and AA genotype of rs711746 should be looked as unfavorable genotypes for individuals’ serum LDL-C level rather than TCHO and TG levels.

### Haplotype analysis for positive SNPs

Based on single site analyses and genotypes’ comparison, haplotypes constructed by rs5803 and rs711746 were analyzed by HBAT program in FBAT software package. Table 3 showed that haplotypes whose distribution and transmission within our family-based population also associated with participants’ serum LDL-C levels (Global *Chi*^2^ = 9.06 *df* = 3, *P* = 0.028). Among three haplotypes, which showed higher frequencies in our sample (Freq. > 5% or Number of informative families > 10), haplotype T-A, especially showed a significant under-transmission correspond to the elevated LDL-C level (*z* = − 2.94, *P*_*- permutation*_ = 0.003). While no significant association were found out by global test between haplotypes, TCHO and TG level (Additional file 1: Table S1).

**Table 3:** Haplotype-base association test between SNPs of *SLC12A3* and LDL-C level

Haplotypes^a^		Freq.	HBAT teststatistics		*P*	*P*_-permutation_^b^	Global *P*
			*Var(S)*	*z*			
*rs5803(T/C)-rs711746(G/A)*							
H1	C-A	0.40	133.45	0.70	0.482	0.486	**0.028** ^c^
H2	C-G	0.38	134.59	1.81	0.071	0.069	
H3	T-A	0.18	93.02	−2.94	**0.003** ^c^	**0.003** ^c^	

*Abbreviations*: *Freq.* frequency of haplotypes, *Var (S)* is a matrix, calculated under the null hypothesis (g.e. no linkage and no association) and used to standardize S

^a^Haplotypes, whose frequencies < 0.05 or number of informative families > 10, were excluded in table and in global *p*-value calculation; ^b^*p*_-permutation_, whole haplotype permutation test (the default time is 10,000); ^c^Bold type denotes *p* < 0.05

### Non-genetic factors effect estimation

To rule out the impact from gender, age, BMI and WHR variables on the relationship between genetic variants and individuals’ LDL-C levels, a correlation analysis was performed within parental group. The age and BMI had a close correlation with individuals’ LDL-C level (*Pearson Correlation* = 0.26, *P* = 0.002 for age; and *Pearson Correlation* = 0.22, *P* = 0.011 for BMI, *Pearson Correlation* = 0.24, *P* = 0.006 for age; and *Pearson Correlation* = 0.17, *P* = 0.050).). While no significant interactions between age, BMI and individuals’ genetic polymorphisms were found (all *P*s > 0.05) (Additional file 1: Table S2).

The statistic power had been calculated by Quanto 1.2.4, that showed more than 0.80 power under dominant or additive genetic models, given a rather lower marginal genetic effect (*R*_g_^2^ = 0.10) averaged over the distribution of the environmental factors.

## Discussion

The amount of lipid and lipoproteins in serum can provide important information about the risk of developing CVD [17]. Among four measurements of serum lipid levels, LDL-C is a well-established risk factor for CVD [18]. Increasing evidences also estimated elevated LDL-C level as a useful indicator for individuals’ hypertension, which is the major cause of CVD amounting 30% of global death rates [19]. To investigate the association between genetic variants and individuals’ serum lipid profiles, four inheritable variables TCHO, TG, HDL-C and LDL-C had been tested and analyzed with a tag-SNPs panel of *SLC12A3* gene. The consistent results of this study showed that, *SLC12A3* gene polymorphism (rs5803 and rs711746) had a close relationship with individuals’ serum LDL-C levels, both in single site and haplotype analysis by FBAT software package (all *P*s < 0.05). These results also revealed the polymorphism of *SLC12A3* gene as an important marker to predict the risk of development CVD, the difference of individuals’ serum LDL-C levels also may be influenced by their genetic background, although the detail mechanism between LDL-C and *SLC12A3* gene still waiting for make clear.

In addition, it needs to be pointed out that as Table 2 described, rs5803 show a significant association with individual’s serum LDL-C level, both under the additive and dominant genetic models (z = − 2.06 *P* = 0.039 for additive model; and z = − 2.08, *P* = 0.037 for dominant model). That is, LDL-C level of CC group will be higher than that of CT + TT groups. Then we used the parental group, at the present study, as a rough random sample to confirm the results of FBAT analysis. Unfortunately, only a distinct difference between CC group and CT + TT group (3.14 mmol/L vs. 2.79 mmol/L, *F* = 0.804, *sig*. = 0.372, *t* = − 1.94 *df* = 131, *P* = 0.055), as showed in Fig. 1. But, we think that this flaw is not enough to negate the association between rs5803 and serum LDL-C level.

At the present study, we also analyzed the association between SNPs and other lipid parameters. As Additional file 1: Table S1 described, from statistical aspect, three SNPs (rs2304478, rs5803 and rs711746) showed association with HDL-C, TCHO and TG levels. However, some of them became negative when corrected by [−e] option (e.g., all *P-*_*TBATe*_ > 0.05, when associated to HDL-C). We also did not observe consistent results from haplotypes analysis (Global *P* = 0.057 for TCHO level; Global *P* = 0.117 for TG level, respectively). Only, the association between SNPs rs5803, rs711746 and LDL-C level were significant and consistent after correction with [−e] option and haplotype global test. So, in this paper, we had introduced the results of LDL-C analysis, mainly.

In general, there are two types of plasma cholesterols, LDL-C and HDL-C, along with one fifth of TG levels make up TCHO, while TG are produced in the liver from excess carbohydrates. LDL-C, makes up the majority of the body cholesterol, and is known as “bad” cholesterol or marker because of its contribution in the development of CVD [20], while HDL-C is considered “good” cholesterol because it helps remove LDL-C from the arteries [21]. The combination of high levels of TG with low HDL-C or high LDL-C cholesterol can increase individuals risk for CVD. So, it was not surprised to observe or in partly reveal association between *SLC12A3* polymorphisms and TG, HDL-C and even TCHO levels. Moreover, a previous research based on European ancestry population revealed the association of*SLC12A3* variants with individuals’ HDL-C level [9]. Our consistent results suggested that, it was LDL-C level, rather than other serum lipid variables, was affected by *SLC12A3* polymorphisms in the Mongolian population. The mechanisms of the association of *SLC12A3*gene with abnormal lipid metabolism have not been elucidated yet. One possible explanation is that *SLC12A3* may indirectly affect blood lipid levels by affecting individual blood pressure. *SLC12A3* gene, encoding the renal thiazide-sensitive Na-Cl cotransporter, is closely related to blood pressure by influencing renal sodium reabsorption, which has been well confirmed by a large number of association studies and functional validation tests [10, 22, 23]. Hypertensive patients are often accompanied by abnormal lipid metabolism; therefore, hypertension and dyslipidemia are closely associated with each other and can affect each other’s morbidity. The second possibility is that recent studies have shown that in addition to hypertension and hyperlipidemia, *SLC12A3* is also associated with type II diabetes [24] and obesity [25]. Several epidemiologic studies have shown that the co-existence of hypertension, and diabetes or obesity is due to common genetic and environmental factors such as diet, physical activity and age [26]. *SLC12A3* may be such a common genetic factor for the above diseases, including dyslipidemia. Of course, the underlying mechanism still needs to clarify.

It is widely accepted that implication for the genetic architecture of hypertension which are major cause of CVD. Furthermore, many alleles that alter renal salt handling in blood pressure variation in the general population [22]. *SLC12A3* gene had been identified and speculated that, its genetic variants and rare mutations will impact the development of hypertension, although more mutations are need to verify [27]. Within Mongolian population, two previous reports also demonstrated the close relationship between *SLC12A3* and hypertension, both with random case-control sample and pedigree cohort [10, 11]. In this study, within parental group which looked as a rough random population, the subjects with homozygous risk alleles also show a higher blood pressure (Additional file 1: Table S3). Considering with the present results, the relationship between *SLC12A3* and CVD became more and more clear that, the genetic variations of *SLC12A3* gene play a vital role in the development of CVD.

Multiple environmental risk factors, including gender, personal fitness status, weight, other physical condition and their interactions, can modulate serum lipid profiles [28, 29], besides genetic background [30, 31]. In the present study, demographic characteristics of participants, including gender, age, BMI and WHR also showed certain influence on individuals’ serum lipid profiles. While the association we observed between *SLC12A3* gene and LDL-C level still significant after controlling these non-genetic factors.

Of course, there are some limitations in this study. Firstly, our study is a single center study and the number of participants included is small, which may affect the persuasion of results. Secondly, in this study, though we checked two health behaviors, including smoking and drinking, however, we didn’t show the related data in the manuscript. There are some reasons as follows: 1) the habits of smoking and drinking are very different among people of different genders and ages. Their influence may be much greater than the effect of the two habits themselves. Moreover, in the family population, because it is not a strict sampling crowd, the crowd cannot control factors such as gender, age, and kinship. If we analyze the effects of smoking and drinking on the levels of lipids in this situation, the credibility of the results will not be satisfactory; 2) our sample size is also limited. If we do a layered analysis, there will be fewer different types of individuals, which will also affect the reliability of the results. Therefore, in this article we did not show the effect of health habits on blood lipid levels. This is also a defect in our article. In short, in this study, we used the pedigree population association analysis method to see that *SLC12A3* polymorphisms are related to LDL-C, but it needs to be further verified in a larger random sample.

## Conclusion

Nevertheless, our results demonstrated the importance of *SLC12A3* polymorphisms in individuals’ difference about their serum lipid profiles, thereby providing evidence for the genetic variants of *SLC12A3* contribution to CVD development via modulating person’s LDL-C level and blood pressure, in certain contexts.

## Additional file

Additional file 1: **Table S1.** The association between tag SNPs of SLC12A3 and TCHO, TG and HDL-C level by FBAT and HBAT methods. **Table S2.** The correlation among blood pressure and lipid parameters. **Table S3.** The influence of rs5803 and rs711746 polymorphism on individuals’ blood pressure. (PDF 147 kb)
