# Supplementary material for: Genetic variants of SLC12A3 modulate serum lipid profiles in a group of Mongolian pedigree population
Source: Lipids Health Dis. 2018 Apr 16;17:83. doi: 10.1186/s12944-018-0737-1 (PMC5902855; doi:10.1186/s12944-018-0737-1)
Supplement: Supplementary file 1 — Table S1. The association between tag SNPs of SLC12A3 and TCHO, TG and HDL-C level by FBAT and HBAT methods. Table S2. The correlation among blood pressure and lipid parameters. Table S3. The influence of rs5803 and rs711746 polymorphism on individuals’ blood pressure. (PDF 147 kb) [file 12944_2018_737_MOESM1_ESM.pdf]

Table S1. The association between tag SNPs of SLC12A3 and TCHO, TG and HDL-C level by FBAT and HBAT methods

Marker [a]	Allele and frequency [b]		Test statistics												
			TCHO				TG				HDL-C			
	Allele	Freq.	*Var* (*S*)	z [c]	p-TBAT	p-TBATe [d]	*Var* (*S*)	z [c]	p-TBAT	p-TBATe [d]	*Var* (*S*)	z [c]	p-TBAT	p-TBATe [d]
rs2304478	G:A	0.14	295.52	-2.26	0.024	-	54.11	-2.03	0.043	0.028	24.02	2.02	0.043	-
rs5803	T:C	0.26	291.21	-2.20	0.028	0.019	66.78	-2.34	0.019	0.020	25.01	-2.18	0.029	-
rs711746	G:A	0.45	400.36	2.24	0.025	0.041	61.78	2.72	0.006	0.042	32.61	2.26	0.024	-

Haplotypes rs5803 (T/C)- rs711746 (G/A) [e]

		Freq.	*Var* (*S*)	z [c]	p-HBATe [f]	Global p [g]	*Var* (*S*)	z [c]	p-HBATe [d]	Global p	*Var* (*S*)	z [c]	p-HBATe [d]	Global p
H1	C-A	0.40	276.98	0.48	-	0.057	36.38	0.57	-	0.117				
H2	C-G	0.38	292.95	1.70	-		44.66	2.15	0.047					
H3	T-A	0.18	197.79	-2.69	0.042		41.07	-2.49	0.034					

Abbreviations: FBAT, Family Based Association Test with the additive model; Freq., frequency of allele or haplotypes constructed by positive SNPs; Var (S), is a matrix, calculated under the null and used to standardize S.

[a] Markers, SNPs and haplotypes showing significant association were demonstrated in this table, and frequency < 0.05 was excluded; [b] alleles and haplotypes detected in more than 10 informative families; and the lower frequency allele was used in following statistics calculation. [c] z-score calculated based on a biallelic marker model for SNPs, positive z values and p < 0.05 indicated a high-risk haplotype; [d] p-TBATe, significance test by FBAT with empirical variance estimator option [-e], non-significant markers displayed with hyphen (-); [e] haplotypes, constructed with SNPs which show positive association consistently; [e] significance test by HBAT with empirical variance estimator option [-e], non-significant haplotypes displayed with hyphen (-); Global p [g], a global test for all haplotypes with frequency > 0.05.

Table S2. The correlation among blood pressure and lipid parameters

	gender	age	SBP	DBP	BMI	WHR	TCHO	TG	HDLC	LDLC
gender		**0.015**	**0.009**	**0.008**	**0.026**	**0.033**	0.681	0.081	**0.005**	0.675
age	-0.21		**<0.001**	**<0.001**	0.210	0.211	**0.034**	0.166	0.770	**0.006**
SBP	-0.23	0.52		**<0.001**	**<0.001**	**0.042**	0.088	0.027	0.111	0.052
DBP	-0.23	0.48	0.89		**<0.001**	**0.014**	0.091	0.029	0.189	0.058
BMI	-0.20	0.11	0.34	0.37		**0.009**	0.340	0.144	0.076	**0.050**
WHR	-0.32	0.19	0.31	0.37	0.393		0.439	0.277	0.701	0.584
TCHO	0.04	0.18	0.15	0.15	0.087	0.120		**<0.001**	**<0.001**	**<0.001**
TG	-0.15	0.12	0.19	0.19	0.133	0.168	0.44		**0.005**	**<0.001**
HDLC	0.24	-0.02	-0.14	-0.12	-0.161	-0.060	0.54	-0.24		**<0.001**
LDLC	0.04	0.24	0.17	0.17	0.178	0.085	0.91	0.33	0.43	

Abbreviations: SBP, systolic blood pressure; DBP, diastolic blood pressure; BMI, body mass index; WHR, waist-hip ratio; TCHO, total plasma cholesterol; TG, triglycerides; HDL-C, high-density lipoprotein cholesterol; LDL-C, low-density lipoprotein cholesterol. The Pearson's correlation coefficient test *r* score displayed in the bottom left, and *P*-value associated with the correlation in the upper right.

Table S3. The influence of rs5803 and rs711746 polymorphism on individuals' blood pressure.

Sample size: n = 129 to 133
Average systolic blood pressure: 140.30±27.93 mmHg
Average diastolic blood pressure: 90.20±15.92 mmHg

	Genotypes groups		t	P
rs5803	CC (n=72)	T carrier (n=63)		
SBP	143.56±26.55	136.68±29.72	1.41	0.161
DBP	92.34±14.63	87.83±17.20	1.63	0.105
rs7114746	AA (n=43)	G carrier (n=86)		

SBP	147.23±28.84	137.54±27.26	1.87	0.064
DBP	94.12±17.28	88.60±15.02	1.87	0.064
